# Identification and verification of grain shape QTLs by SNP array in rice

**DOI:** 10.1371/journal.pone.0260133

**Published:** 2021-11-22

**Authors:** Junxiao Chen, Kai Liu, Wenjun Zha, Lei Zhou, Ming Li, Huashan Xu, Peide Li, Zhijun Chen, Guocai Yang, Pingli Chen, Sanhe Li, Aiqing You

**Affiliations:** 1 Hubei Key Laboratory of Food Crop Germplasm and Genetic Improvement, Food Crops Institute, Hubei Academy of Agricultural Sciences, Wuhan, China; 2 Guangdong Key Laboratory of New Technology in Rice Breeding, The Rice Research Institute of Guangdong Academy of Agricultural Sciences, Guangzhou, China; The Institute of Genetics and Developmental Biology (IGDB) of the Chinese Academy of Sciences (CAS), China, CHINA

## Abstract

Grain shape strongly influences the economic value and grain yield of rice. Thus, identifying quantitative trait loci (QTLs) for grain shape has been a longstanding goal in rice genetic research and breeding programs. Single nucleotide polymorphism (SNP) markers are ubiquitous in the rice genome and are more abundant and evenly distributed on the 12 rice chromosomes than traditional markers. An F_2_ population was genotyped using the RICE6K SNP array to elucidate the mechanisms governing grain shape. Thirty-five QTLs for grain shape were detected on 11 of 12 chromosomes over 2 years. The major QTL cluster *qGS7* was detected in both years and displayed strong genetic effects on grain length and width, showing consistency with *GL7/GW7*. Some minor QTLs were also detected, and the effects of four QTLs on seed size were then validated using BC_1_F_6_ populations with residual heterozygous lines in each QTL region. Our findings provide insights into the molecular basis of grain shape as well as additional resources and approaches for producing hybrid high-yield rice varieties.

## Introduction

It is critical to increase crop productivity through efficient breeding to meet the challenge of feeding a rapidly growing world population in a smaller area [1]. Rice (*Oryza sativa*) is a staple food crop worldwide and a key model cereal crop. Grain shape in rice is controlled by a combination of grain length, grain width, and grain thickness and is a major determinant of grain yield and grain appearance quality [2]. Therefore, it is essential to explore the genetic basis of grain shape and identify new quantitative trait loci (QTLs) for the improvement of grain yield and quality.

Most QTLs influence grain shape by regulating cell division or proliferation, and several major QTLs have been cloned and functionally characterized. *GS3*, the first cloned gene for grain shape, encodes a putative transmembrane protein and function as a negative regulator of grain length [3, 4]. The QTL *qGL3/qGL3*.*1* encodes a protein phosphatase with a Kelch-like repeat domain and negatively controls grain length [5]. For grain width, *GW2* encodes a novel RING-type protein with E3 ubiquitin ligase activity and regulates grain width [6]. *GW5/GSE5* codes for a plasma membrane-associated protein and governs grain width; a deletion in the promoter region causes decreased expression and increases grain width [7, 8]. *GS5* encodes a putative serine carboxypeptidase and is a positive regulatory factor of grain size [9], and the *GS5* allele with a higher expression level resulted in increased grain size. Similarly, *GW8*, which encodes the transcription factor *OsSPL16* with an SBP domain, is also a positive regulator of grain size [10]. *GL7/GW7*, contains a 17.1-kb tandem duplication and encodes a protein homologous to *Arabidopsis thaliana* LONGIFOLIA proteins, increases grain length and reduces grain width [11, 12]. The cloning and functional characterizations of these genes have greatly enriched our knowledge of the molecular mechanisms determining grain shape and improve genomic breeding in rice [13, 14]. However, additional genes with minor effects on grain shape remain largely unknown.

QTL mapping is frequently used in genetic studies to identify new genes and QTLs. Compared with traditional molecular markers, single nucleotide polymorphisms (SNPs) are more abundant and uniformly distributed on the 12 rice chromosomes and are regarded as the most desirable molecular marker [15]. Next-generation sequencing technologies have identified millions of DNA sequences, improving the discovery of SNPs [16, 17]. Whole-genome sequencing and DNA microarrays [18] are high-throughput genotyping platforms for constructing high-density genetic linkage maps with SNPs. Both methods have been applied in gene discovery [19–21], and microarrays have been widely used in studies of humans, *Arabidopsis*, and rice to produce high-resolution linkage maps [22–24].

This study constructed a high-resolution genetic linkage map using a high-density SNP array. Linkage analyses were performed in F_2_ and its derived F_3_ populations to determine the genetic basis of grain size in rice. Our results provide insights into the construction of linkage maps with gene arrays, and the identified QTLs can potentially improve rice grain size.

## Materials and methods

### Plant materials and field planting

**Fig 1:**
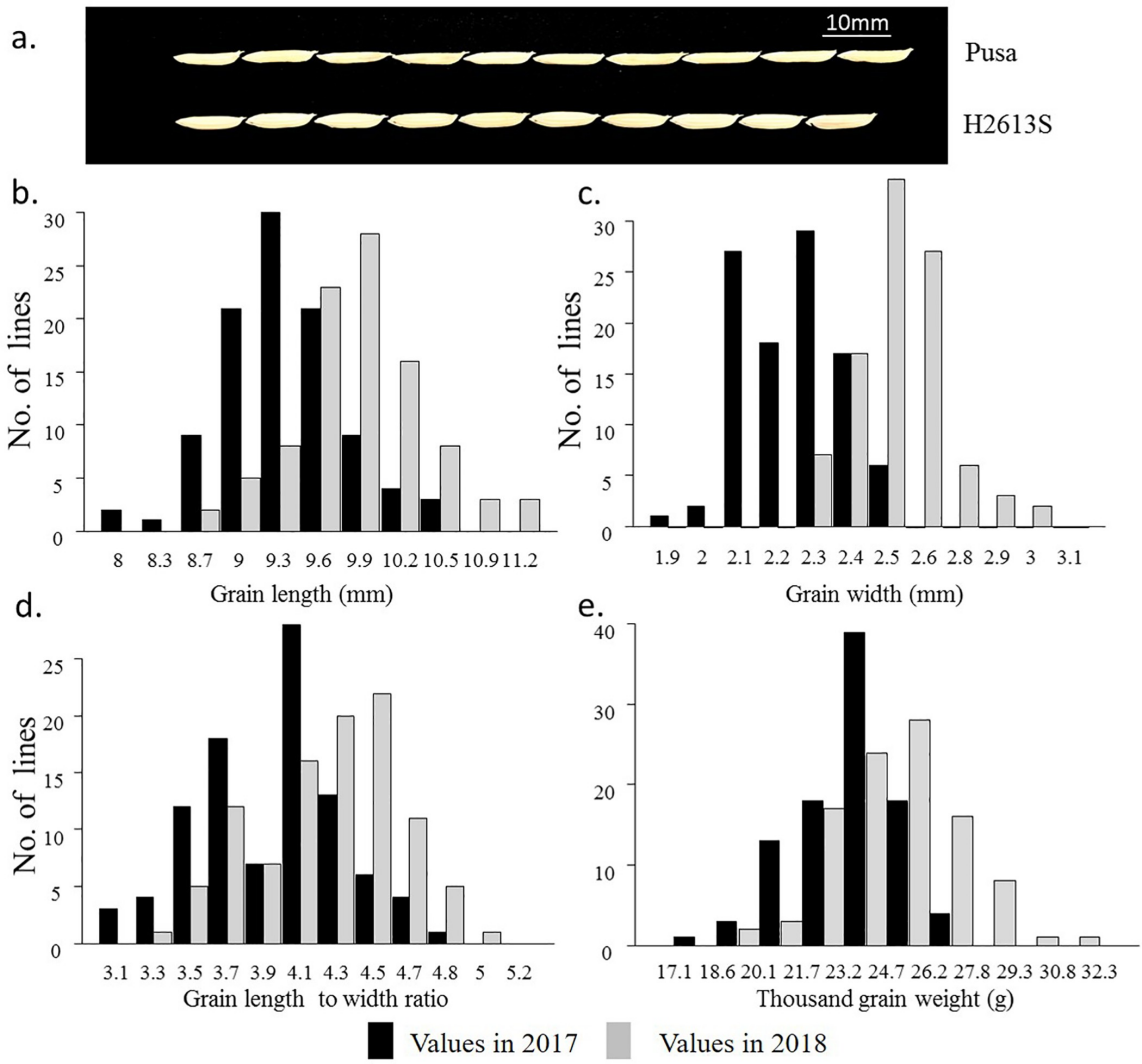
Frequency distribution of grain length, grain width, grain length-to-width ratio, and thousand-grain weight of the F_2_ and its derived F_3_ populations in 2017 and 2018, respectively.

An F_2_ population was constructed by crossing the long-grain variety, *O*. *sativa* cv. Pusa, with the medium-grain variety, *O*. *sativa* cv. H2613S. The male parent (Pusa) has the longest grain in a collection of 529 core germplasms. The female parent H2613S is a well-known male sterile line characterized by excellent plant architecture and several advantages regarding quality and resistance (Fig 1A). Five hundred F_2_ plants were obtained from the cross between H2613S and Pusa. Two markers closely linked to the male sterile gene *tms5* (TMS1F: AAGTTGCCACCCTCTTTCAG, TMS1R: TGTGTGAAGGGGTGCTACAG; TMS2F: GCAAAAGCTCAAGCCAGAGT, TMS2R: TCTCAGGCACCGTCAATGTA) were used to detect the fertility of these F_2_ lines. One hundred fertile plants with a homozygous genotype at the *tms5* locus were used as the mapping population. The F_2_ population was planted in 2017, and the F_3_ population derived from the F_2_ population was planted in 2018, during normal growing seasons at the experimental field in Wuhan, China. H2613S was used as the recurrent parent to construct the backcross BC_1_F_1_ populations. Then, four respectively derived plants in the BC_1_F_1_ were self-pollinated to obtain BC_1_F_2_ populations. BC_1_F_6_ residual heterozygous lines were generated using seed descent and marker-assisted selection and were planted in 2020 under the same experimental conditions to confirm the effects of QTLs. All accessions were sown in the seedling nursery, and 25-day-old seedlings were transplanted into two-row plots (12 plants, with 26.5 cm of space between the rows and 16.5 cm of space between plants within a row).

### Trait measurements and statistical analyses

Harvested rice grains were air-dried and stored at room temperature for at least 3 months, and all fully-filled rice grains were collected from each plant to investigate grain shape traits, including grain length, grain width, grain length-to-width ratio, and thousand-grain weight. These traits were measured at a high-throughput rice phenotyping facility [25]. *P*-values for phenotypic coefficients were calculated using a two-sided *t-*test and the cor.test function in R software [26].

### Genotyping and linkage map construction

The F_2_ population was genotyped using the RICE6K array, which contained 5,102 SNP and InDel markers. There were 1,596 unique variants between Pusa and H2613S (S1 Fig). The genetic linkage map of the population was calculated using the MapMaker 3.0 program. Analyses of main-effect QTLs were conducted in the mapping population by composite interval mapping using WinQTLCart version 2.5. In the analyses, the likelihood ratio and *t-*test were combined to test the significance of the single-locus QTL additive effects. The likelihood ratio and *t* values corresponding to *P* = 0.001, equivalent to LOD (log likelihood value) ≥ 3.0, were used as the threshold for selecting the putative main-effect QTLs. The peak points of the likelihood ratio in the linkage map were considered the putative positions of the QTLs. The relative contribution of a genetic component (%) was calculated as the proportion of phenotypic variance explained by that component in the selected model [27].

## Results

### Grain shape variation in the F_2_ and its derived F_3_ populations

The phenotypic values of grain length and width were both higher in 2018 than in 2017 (Fig 1B–1D). The grain length and thousand-grain weight were normally distributed and showed a continuous variation in both years (Fig 1B and 1E), and grain width also showed a continuous variation and approximately followed a normal distribution (Fig 1C). The distribution of the length-to-width ratio was bimodal in both years, and the two peaks were close to each other (Fig 1D).

Data on grain length, width, length-to-width ratio, and thousand-grain weight are shown in Tables 1 and 2. These phenotypes were strongly correlated with each other, except thousand-grain weight and grain length-to-width ratio. Grain width was significantly and negatively correlated with grain length and thousand-grain weight was significantly and positively associated with grain length and grain width in both years.

**Table 1:** Grain Length (GL), Grain Width (GW), grain Length-to-Width Ratio (LWR), and Thousand-Grain Weight (TGW) of the F_2_ and its derived F_2:3_ populations in 2017 and 2018, respectively.

	GL17	GL18	GW17	GW18	LWR17	LWR18	TGW17	TGW18
	(mm)	(mm)	(mm)	(mm)			(g)	(g)
Mean	9.32	9.90	2.28	2.57	3.95	4.21	22.64	25.77
SD	0.46	0.51	0.14	0.13	0.35	0.36	1.77	2.20
Minimum	8.12	8.71	1.95	2.28	3.20	3.30	17.20	19.50
Maximum	10.57	11.26	2.59	3.01	4.90	5.10	26.93	32.44

GL17, grain length in 2017; GW17, grain width in 2017; LWR17, grain length to width ratio in 2017; TGW17, thousand grain weight in 2017; GL18, grain length in 2018; GW18, grain width in 2018; LWR18, grain length to width ratio in 2018; TGW18, thousand grain weight in 2018

**Table 2:** Correlation coefficients among grain shape traits of the F_2_ and its derived F_2:3_ populations in 2017 and 2018, respectively.

*Correlation*	GL17	GL18	GW17	GW18	LWR17	LWR18	TGW17	TGW18
	(mm)	(mm)	(mm)	(mm)			(g)	(g)
GL17								
GL18	0.79**							
GW17	–0.20**	–0.39**						
GW18	–0.26**	–0.43**	0.64**					
LWR17	0.63**	0.85**	–0.60**	–0.82**				
LWR18	0.70**	0.73**	–0.83**	–0.60**	0.78**			
TGW17	0.47**	0.25**	0.50**	0.28**	–0.02	–0.11		
TGW18	0.36**	0.32**	0.24**	0.46**	–0.09	0.03	0.58**	

*Significant at *P* < 0.01

** Significant at *P*< 0.001.

### Mapping of QTLs for grain shape

**Fig 2:**
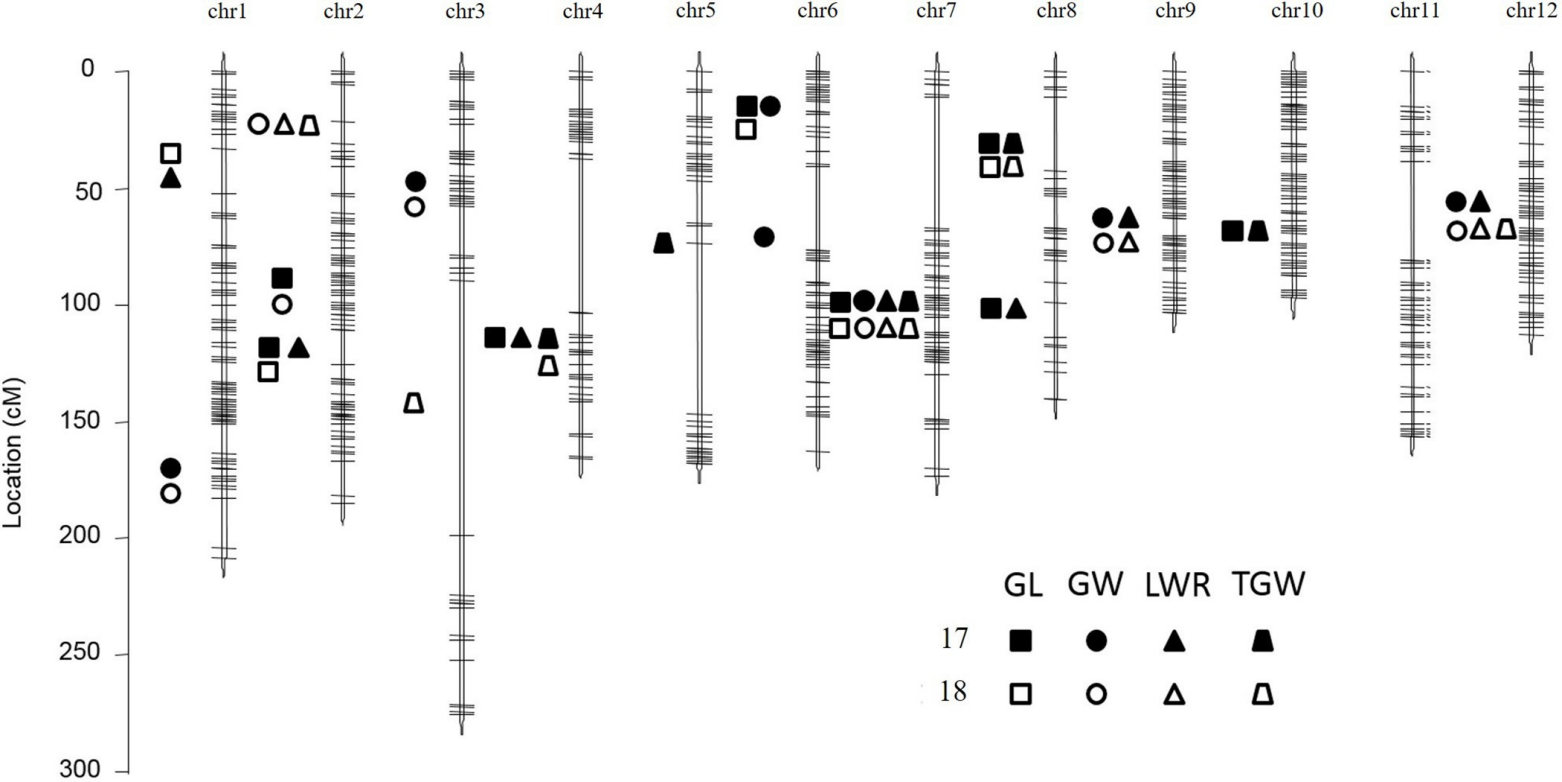
Distribution of putative QTLs for grain shape traits on the linkage map. GL, grain length; GW, grain width; LWR, grain length-to-width ratio; TGW, thousand-grain weight.

LOD thresholds for selecting QTLs for grain shape traits were determined by 1000 permutations at *P* = 0.05. Thirty-five QTLs for grain shape traits were distributed on all chromosomes, except chromosome 11, in the two years (Table 3 and Fig 2). The phenotypic variance explained by each QTL ranged from 0.12% to 74.26%.

**Table 3:** Putative QTLs for grain shape traits detected in two years.

QTLs	Chr	Interval	2017			2018		
			LOD	Add	PVE	LOD	Add	PVE
*qGL1*	1	R0103700–F0105565				3.00	0.17	1.81%
*qGL2*.*1*	2	F0221402–R0223696	2.94	-0.16	3.85%			
*qGL2*.*2*	2	F0224762–R0233798	3.22	-0.15	7.23%	2.00	-0.21	4.56%
*qGL4*	4	F0422786–F0428307	2.73	0.10	0.57%			
*qGL6*	6	F0600400–F0603241	3.21	-0.16	3.85%	2.48	-0.13	3.76%
*qGL7*	7	R0721009–R0726296	16.71	0.45	59.20%	12.11	0.50	52.84%
*qGL8*.*1*	8	R0809627–F0820874	3.63	-0.21	3.89%	2.93	-0.12	0.12%
*qGL8*.*2*	8	F0821752–F0823314	2.93	0.06	9.24%			
*qGL10*	10	R1017809–R1018706	2.25	0.14	0.23%			
*qGW1*	1	R0130317–0142922	3.08	-0.05	2.78%	2.00	-0.02	1.58%
*qGW2*.*1*	2	F0204975–R0207632				6.34	0.09	26.70%
*qGW2*.*2*	2	F0220300–R0223696				4.65	-0.07	2.88%
*qGW3*	3	F0305442–F0314197	2.30	-0.04	10.96%	4.20	-0.06	17.29%
*qGW6*.*1*	6	F0600400–R0604673	4.93	-0.06	5.97%			
*qGW6*.*2*	6	F0605650–F0617668	3.20	0.01	5.74%			
*qGW7*	7	R0721009–R0726296	15.62	-0.14	46.81%	11.52	-0.13	30.97%
*qGW9*	9	R0906011–F0918471	4.58	-0.05	11.80%	3.39	-0.06	7.19%
*qGW12*	12	R1204910–R1223769	4.95	-0.06	5.29%	2.69	-0.05	6.25%
*qLWR1*	1	R0108168–R0110660	3.03	0.02	2.37%			
*qLWR2*.*1*	2	F0204975–R0207232				3.70	-0.14	13.54%
*qLWR2*.*2*	2	F0224762–F0229600	3.11	-0.10	4.39%			
*qLWR4*	4	F0422786–F0427798	4.01	0.02	0.99%			
*qLWR7*	7	F0721726–R0726296	30.75	0.46	74.26%	15.13	0.37	44.96%
*qLWR8*	8	F0805716–R0820139	3.15	-0.09	2.21%			
*qLWR9*	9	R0906011–R0919382	3.34	-0.07	5.49%	3.95	-0.15	2.41%
*qLWR12*	12	R1203509–R1207724	2.40	0.13	8.36%	4.38	0.15	9.63%
*qTGW2*	2	F0202110–R0206070				4.10	0.92	23.09%
*qTGW3*	3	R0326839–R0331832				2.90	0.45	12.36%
*qTGW4*	4	R0416449–F0430948	4.61	1.01	6.80%	3.34	0.64	6.56%
*qTGW5*	5	R0514657–R0529509	5.14	1.01	23.64%			
*qTGW7*.*1*	7	F0705336–F0716437				3.35	0.24	2.57%
*qTGW7*.*2*	7	R0710169–F0721726	2.41	0.41	10.52%	3.66	0.29	14.47%
*qTGW8*	8	R0801125–R0809627	2.95	-0.77	3.26%	3.08	-0.69	2.68%
*qTGW10*	10	F1012057–R1018706	6.14	-1.00	18.26%			
*qTGW12*	12	R1224193–R1226674				2.86	-0.79	10.62%

Add, the additive effect of each QTL; PVE, the phenotypic variance explained by each QTL; LOD, logarithm of odds; *qGL*, QTL for grain length; *qGW*, QTL for grain width; *qLWR*, QTL for grain length-to-width ratio; *qTGW*, QTL for thousand-grain weight.

For grain length, a major QTL was detected on chromosome 7. The QTL (*qGL7*) was flanked by R0721009 and R0726296 and was stabled in both years; it explained 59.20% of the phenotypic variation in 2017 and 52.84% in 2018. In addition, eight minor QTLs (*qGL2*.*2*, *qGL6*, *qGL8*.*1*, *qGL1*, *qGL2*.*1*, *qGL4*, *qGL8*.*2*, and *qGL10*) were detected on chromosomes 1, 2, 4, 6, 8, and 10. Of these, *qGL2*.*2*, *qGL6*, and *qGL8*.*1* were detected in both years and explained 0.12% to 7.23% of the phenotypic variation, and *qGL1*, *qGL2*.*1*, *qGL4*, *qGL8*.*2*, and *qGL10* were detected in one year and explained 0.23% to 9.24% of the phenotypic variation.

For grain width, a major QTL was detected on chromosome 7 in the two years. The QTL (*qGW7*) was flanked by R0721009 and R0726296 and explained 46.81% of the phenotypic variation in 2017 and 30.97% in 2018. Moreover, eight minor QTLs (*qGW1*, *qGW3*, *qGW9 qGW1*2, *qGW2*.*1*, *qGW2*.*2*, *qGW6*.*1*, and *qGW6*.*2*) were detected on chromosomes 1, 2, 3, 6, 9, and 12. Of these, *qGW1*, *qGW3*, *qGW9*, and *qGW1*2 were detected in both years and explained 1.58% to 17.29% of the phenotypic variation, and *qGW2*.*1*, *qGW2*.*2*, *qGW6*.*1*, and *qGW6*.*2* were detected in one year and explained 2.88% to 26.70% of the phenotypic variation.

Marker-trait linkage analyses revealed that eight QTLs for the grain length-to-width ratio were detected on chromosomes 1, 2, 4, 7, 8, 9, and 12. Among them, the QTL flanked by F0721726 and R0726296 on chromosome 7 (*qLWR7*) was found in the two years, and explained 74.26% of the phenotypic variation in 2017 and 44.96% in 2018. *qLWR12* was flanked by R1203509 and R1207724 on chromosome 12 and was detected in both years. This QTL explained 8.36% of the phenotypic variation in 2017 and 9.63% in 2018. Moreover, one and five QTLs were detected in both years and one year, respectively; these explained 0.99% to 13.54% of the phenotypic variation.

Nine QTLs for thousand-grain weight were detected on chromosomes 2, 3, 4, 5, 7, 8, 10, and 12. Three minor QTLs were detected in both years and explained 2.68% to 14.47% of the phenotypic variation. The remaining QTLs were detected in one year and explained 2.57% to 23.64% of the phenotypic variation.

### High precision of QTL mapping via high-density SNP array

**Fig 3:**
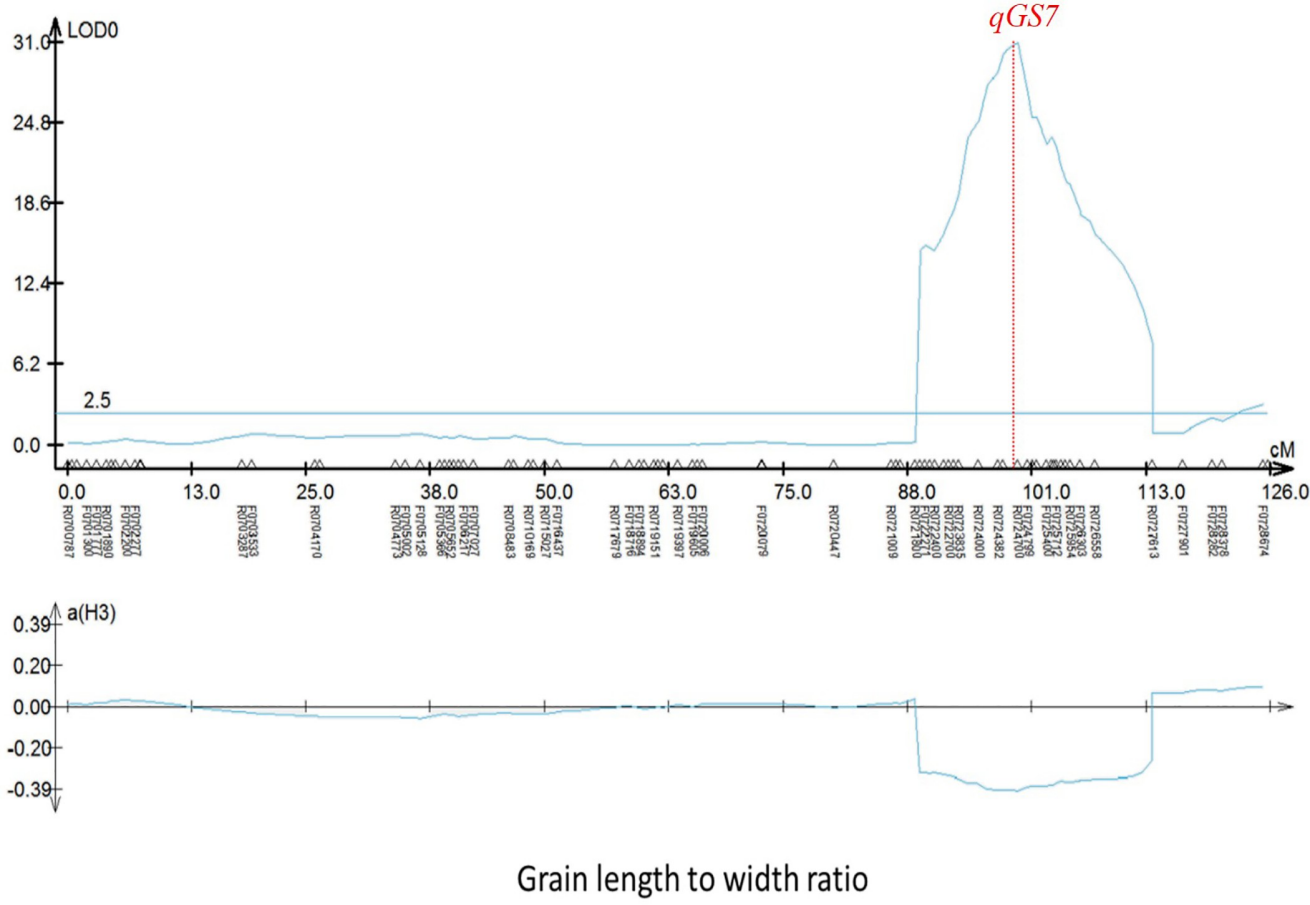
Local *qGS7* mapping result of grain width. Red line indicates the position of the peak SNP.

The region flanked by R0710169 and R0726296 on chromosome 7 contained four QTLs, *qGL7*, *qGW7*, *qLWR7*, and *qTGW7*.*2*, which were collectively termed *qGS7*. These QTLs controlled grain length, grain width, grain length-to-width ratio, and thousand-grain weight, respectively. The top points of *qGL7*, *qGW7*, and *qLWR7* were all located between R0724564 and R0724700, corresponding to 136 kb (Fig 3). According to the Nipponbare 6.0 reference genome, 15 candidate genes are located in this 136-kb region, including a major grain size gene, *GL7/GW7*. Some minor candidate QTLs possessing gene linkage or pleiotropy were observed as well. The QTL *qGW12* was identified in both years, and the top point was located between R1220728 and R1221279, corresponding to 551 kb (S2 Fig). Exploring and utilizing the linkage or pleiotropy of these QTLs underlying grain traits would be beneficial for genetic research and grain yield improvement. These results support the high precision of QTL mapping via high-density SNP arrays.

### Validation of the genetic effects of major and minor QTLs

**Fig 4:**
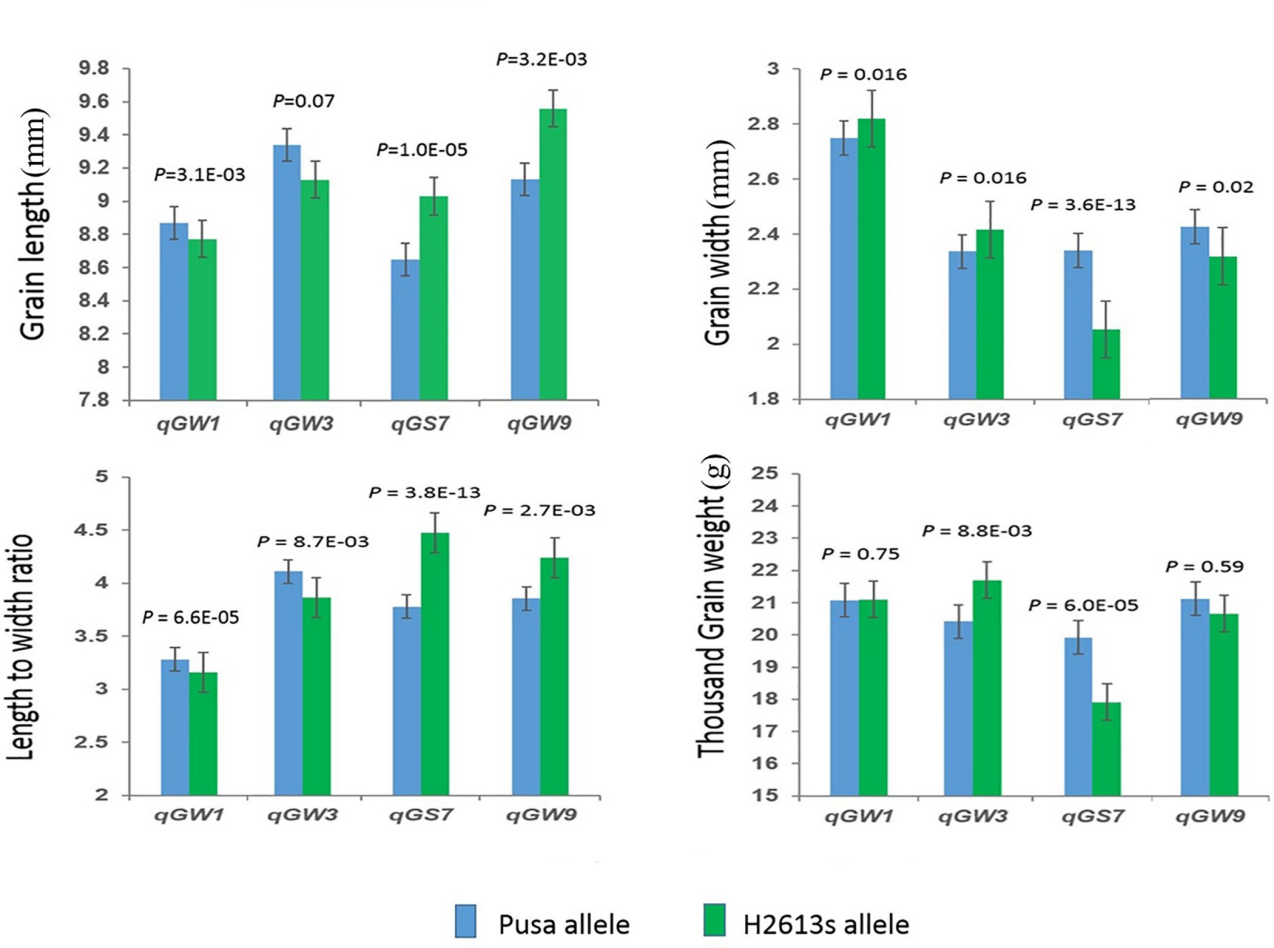
Genetic effects of *qGW1*, *qGW3*, *qGS7*, and *qGW9* on grain length, grain width, grain length-to-width ratio, and thousand-grain weight. The BC_1_F_6_ residual heterozygous line populations of each QTL were analyzed to validate the genetic effects of these QTLs. Blue bars represent alleles from pusa, Green bars represent alleles from H2613s. Error bars are based on standard deviation of each genotype. *P*-values based on two-tailed t-test.

The BC_1_F_6_ residual heterozygous line populations of *qGW1*, *qGW3*, *qGS7*, and *qGW9* were analyzed to validate the genetic effects of these QTLs (Fig 4). The *qGW1* locus from female parents increased grain width by 0.05 mm. The *qGW3* locus from female parents increased grain width by 0.04 mm and had little effect on grain length. The *qGS7* locus from male parents increased grain length by 0.20 mm and decreased grain width by 0.15 mm. All these QTLs had significant effects on grain length-to-width ratio. These findings suggest that these QTLs have great potential for grain size improvement.

## Discussion

Rice is a staple food, feeding more than half of the world’s population [1]. Rice grain shape is one of the most important factors contributing to rice yield. Therefore, the identification of major or minor QTLs for grain shape is a longstanding goal in rice genetic research and breeding programs. In this study, a RICE6K SNP array with high-density SNP markers was used in genotyping to identify QTLs for grain shape traits.

Grain size and grain weight are agronomic traits with high heritability in rice. However, the phenotypic values of grain length, grain width and grain weight were all lower in 2017 than in 2018 (Fig 1) because these quantitative traits are largely affected by the environment. Ambient temperature was higher in the summer of 2017 than in the summer of 2018, potentially affecting grain filling. Genetic factors may also contribute to phenotypic differences between the two study years because the dominance effect was higher in F_2_ than F_3_.

In the present study, 35 QTLs for grain shape were detected in two years. Among them, a QTL cluster on chromosome 7 contributed to grain length and grain width and displayed strong genetic effects, and the *qGS7* allele tended to increase grain length and decrease grain width. This region also contained the gene *GL7*/*GW7*, cloned previously [11, 12]. *GL7*/*GW7* encodes a TONNEAU1-recruiting motif protein and acts as a semi-dominant locus. Upregulation of *GL7*/*GW7* expression leads to a decrease in cell width and an increase in cell length of epidermal cells of the inner and outer glumes. In our study, the genomic DNA fragments from the candidate gene in Pusa and H2613S were sequenced and analyzed using primers NGSP11F and 210QCF, and a 1,471 bp fragment with a functional allele associated with longer grain length was amplified from the male parent, consistent with previous results (S3 Fig). Thus *GL7*/*GW7* might be the candidate gene for *qGS7*.

Several minor QTLs for grain shape appeared as novel QTLs that have not been reported previously. In both years, the *qGL2*.*2* region was close to the SNP marker KASP0224 and showed moderate effects on grain length and grain width. The *qGW9* region was close to the SNP marker KASP0915 and was responsible for grain width and length-to-width ratio in both years. Similarly, *qGW1* was identified in both years and affected on grain width. These regions contained several minor QTLs for grain width, consistent with our previous study in other populations. *GS3* and *GW5* are two major genes respectively for grain length and grain width, and strongly contribute to grain length and width variance in rice landraces [28]. However, these genes were not identified in QTL mapping in our samples, probably because the genotypes of *GS3* and *GW5* in the two parental lines were identical.

Since the sequencing of two rice genome references (93–11 and Nipponbare), millions of SNPs are currently available on the NCBI website and are used as genetic markers. Compared with traditional markers, such as RFLP and SSR, SNPs are ubiquitous in the rice genome and are highly abundant and evenly distributed on the 12 rice chromosomes, improving the development of linkage maps using RFLP and SSR markers. In addition, SNP genotyping using a SNP array is cheaper than whole-genome sequencing and can efficiently obtain the genotypes of genome-wide SNPs for several individuals from an experimental population [29]. Nonetheless, the development of high-quality linkage maps is limited by the population size and number of markers. Thus, the resolution of QTL mapping is positively correlated with the density of the genetic map and population size [30], and the number of polymorphic markers evaluated in QTL analyses increases as the use of SNP arrays expands. In our study, the top SNPs (*qGL7*, *qGW7*, and *qLWR7*) were located between R0724564 and R0724700, a span of 136 kb, and this region is highly consistent with *GL7*/*GW7*. These studies suggest that SNP arrays are an efficient and reliable genotyping method to identify new genes or QTLs.

Most rice cultivars approved in China show medium grain size, good quality, and high yield; nonetheless, these characteristics can be improved in many cultivars [31]. Therefore, breeding rice cultivars with a combination of desirable traits is challenging. Although some genes have been cloned and many QTLs have been identified in rice cultivars, the genetic basis and molecular mechanism underlying grain shape and quality are incompletely understood [32]. A previous study showed that mature grains of the near-isogenic line NIL-*gs9* were slender than those of Nipponbare, and the transparency of mature rice was greatly improved in NIL-*gs9*, which showed no or very little chalkiness [33]. The authors designed QTL pyramiding based on combinations of alleles *qgw8* and *qgs3* by molecular marker-assisted selection and developed a slender rice variety, substantially improving grain quality [10]. These results suggest that grain width may be correlated with the degree of chalkiness. The major QTL *qGS7* can potentially produce slender and less chalky grains. The QTL *qGW3* affected grain width but not grain length, potentially influencing chalkiness. *qGW9* had a strong effect on grain length but a slight effect on grain width. Additional characterization and validation studies combined with fine mapping are necessary to identify novel genes and alleles and improve our understanding of the relationship between these candidate genes and the observed phenotypes, providing researchers with effective genetic tools to select beneficial alleles.

## Supporting information

S1 Fig Haplotype maps of example lines/plants detected by RICE6K array. (a) One Pusa/H2613S F_2_ line. (b) The whole Pusa/H2613S F_2_ lines. Each short line at the chromosomes indicates the position of a single nucleotide polymorphism (SNP); AA, female parental homozygous genotype; BB, male parental homozygous genotype; and AB, heterozygous genotype.
(TIF)

S2 Fig Local *qGW12* mapping result of grain width. Red line indicates the position of the peak SNP.
(TIF)

S3 Fig The gel image of the PCR amplification with the 210QCF and NGSP11F primer pair using DNA samples from Pusa, H2613s and C815s. (TIF)

S1 Table List of primers used in this study. (DOCX)

S2 Table The genotyping results of *qGW1*, *qGW3*, *qGS7* and *qGW9* in the BC_1_F_6_ residual heterozygous lines. (XLSX)
