# Supplementary figures and images for: Identification and verification of grain shape QTLs by SNP array in rice
Source: PLoS One. 2021 Nov 22;16(11):e0260133. doi: 10.1371/journal.pone.0260133 (PMC8608341; doi:10.1371/journal.pone.0260133)

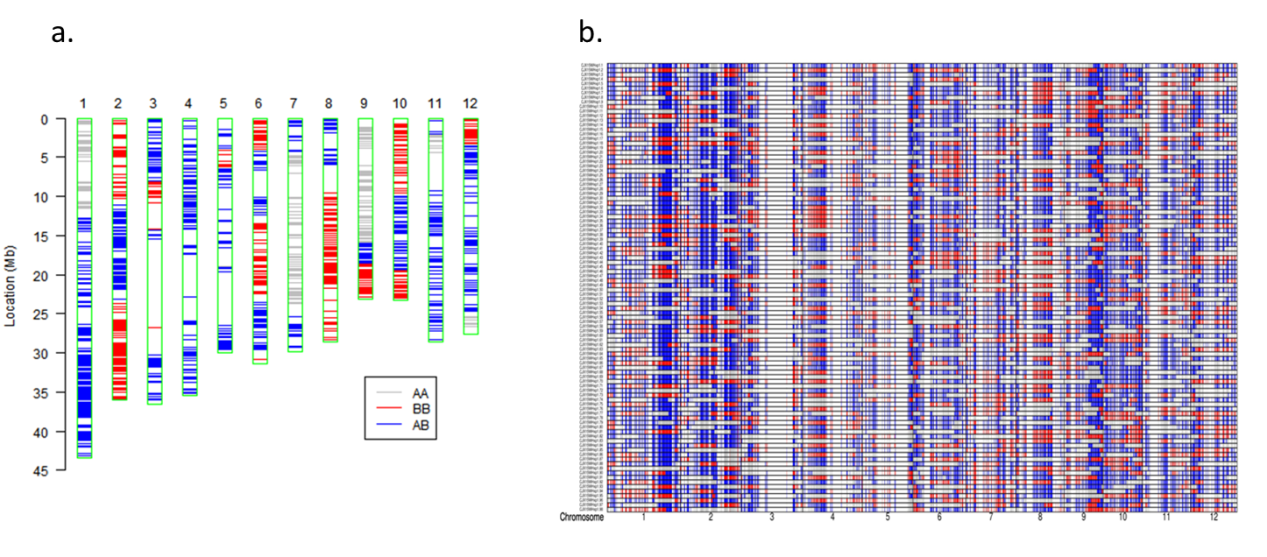

Supplement: S1 Fig — (a) One Pusa/H2613S F2 line. (b) The whole Pusa/H2613S F2 lines. Each short line at the chromosomes indicates the position of a single nucleotide polymorphism (SNP); AA, female parental homozygous genotype; BB, male parental homozygous genotype; and AB, heterozygous genotype. (TIF) [file pone.0260133.s001.tif]

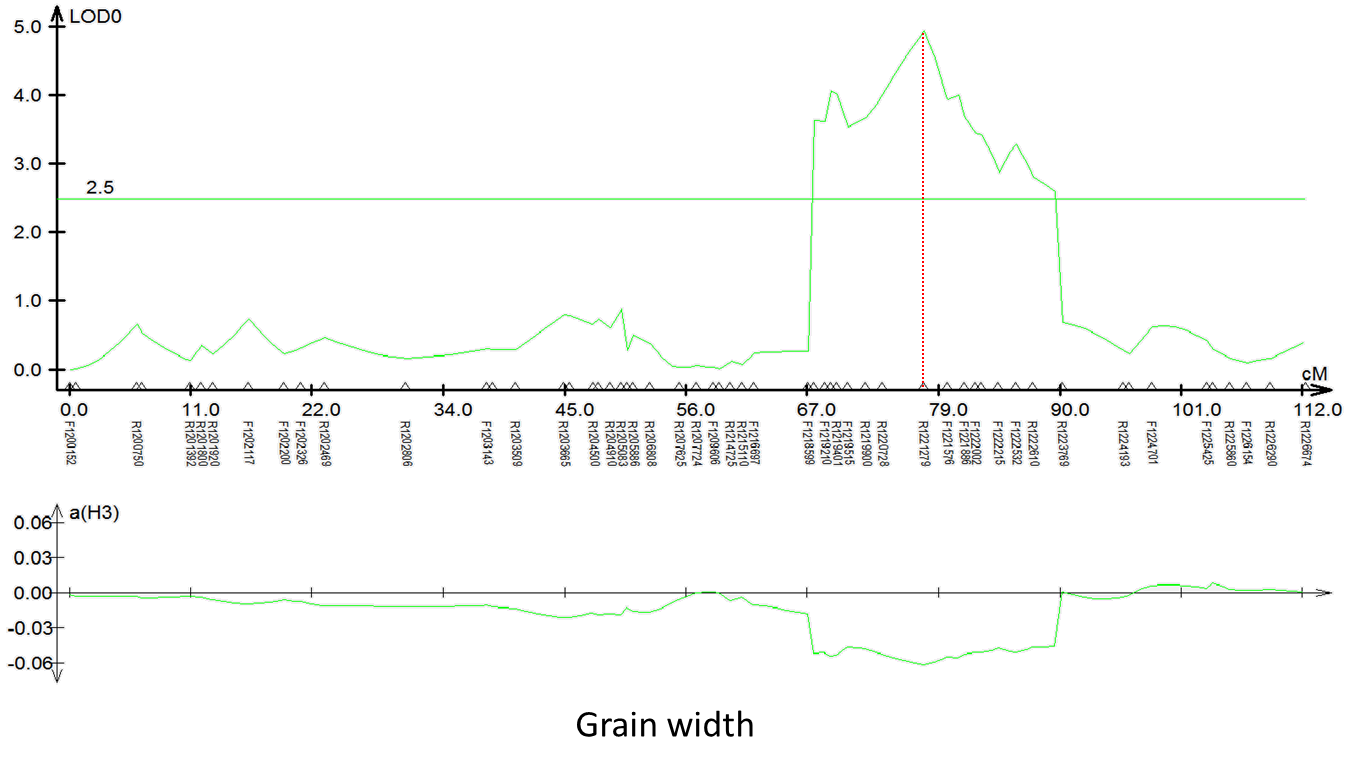

Supplement: S2 Fig — Red line indicates the position of the peak SNP. (TIF) [file pone.0260133.s002.tif]

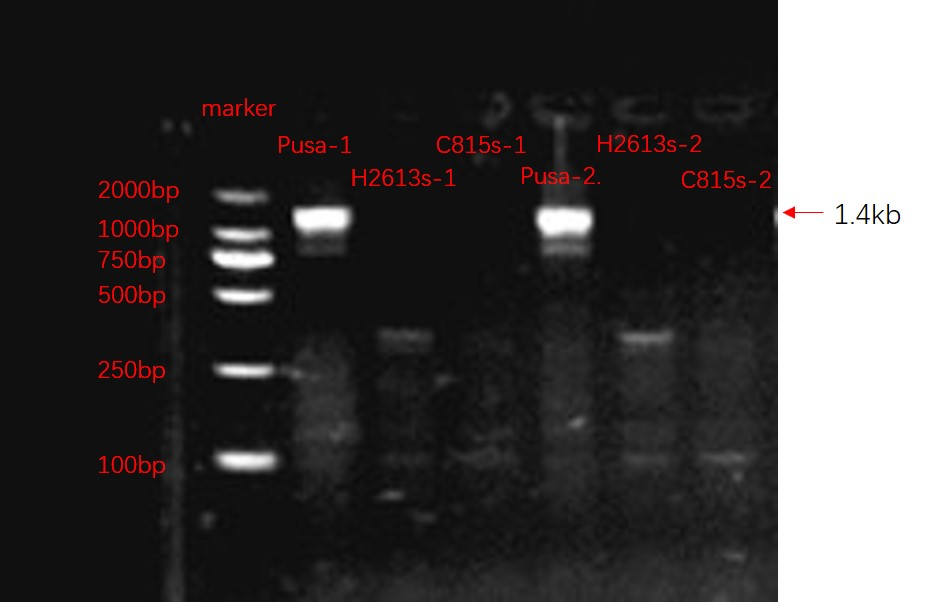

Supplement: S3 Fig — (TIF) [file pone.0260133.s003.tif]
