# Supplementary material for: Identification and verification of grain shape QTLs by SNP array in rice
Source: PLoS One. 2021 Nov 22;16(11):e0260133. doi: 10.1371/journal.pone.0260133 (PMC8608341; doi:10.1371/journal.pone.0260133)
Supplement: S1 Table — (DOCX) [file pone.0260133.s004.docx]

**S1 Table. List of primers used in this study**

| primer | sequence(5′-3′) |
| --- | --- |
| KASP01418F1 | GAAGGTGACCAAGTTCATGCTTGAGTTTACTTCTTATTTGA |
| KASP01418F2 | GAAGGTCGGAGTCAACGGATTTGAGTTTACTTCTTATTTGC |
| KASP01418R | CCTACAATGGCTGGAGTAGGA |
| KASP01428F1 | GAAGGTGACCAAGTTCATGCTCTCTCTCACTGCAAAAGGCG |
| KASP01428F2 | GAAGGTCGGAGTCAACGGATTCTCTCTCACTGCAAAAGGCA |
| KASP01428R | ATGCTGTCAAATAAGGTTGG |
| KASP02214F1 | GAAGGTGACCAAGTTCATGCTGTGCTCCTCCATGATCTTGT |
| KASP02214F2 | GAAGGTCGGAGTCAACGGATTGTGCTCCTCCATGATCTTGC |
| KASP02214R | CATGCCAATCTCATTGGAGG |
| KASP0224F1 | GAAGGTGACCAAGTTCATGCTAGCGCAATAACCGGCATTCT |
| KASP0224F2 | GAAGGTCGGAGTCAACGGATTAGCGCAATAACCGGCATTCC |
| KASP0224R | CAAGTCGCAGGCAGTAGCTT |
| KASP02274F1 | GAAGGTGACCAAGTTCATGCTAATCTTCACACGTATCATGC |
| KASP02274F2 | GAAGGTCGGAGTCAACGGATTAATCTTCACACGTATCATGT |
| KASP02274R | CATTCAACGATGCTTTGGTT |
| KASP03055F1 | GAAGGTGACCAAGTTCATGCTCGTCGCCGGCAATGGTCGCA |
| KASP03055F2 | GAAGGTCGGAGTCAACGGATTCGTCGCCGGCAATGGTCGCG |
| KASP03055R | GCCATGGTCAGATGATGATC |
| KASP03083F1 | GAAGGTGACCAAGTTCATGCTGTTCGGGTGGTCTAACCGGA |
| KASP03083F2 | GAAGGTCGGAGTCAACGGATTGTTCGGGTGGTCTAACCGGC |
| KASP03083R | CTGCCGTCGGTCAGACCACC |
| KASP0723835F1 | GAAGGTGACCAAGTTCATGCTGTTAATTCTCTTCTAAAATT |
| KASP0723835F2 | GAAGGTCGGAGTCAACGGATTGTTAATTCTCTTCTAAAATC |
| KASP0723835R | GACATGAACAGTAACCTGCA |
| KASP07253F1 | GAAGGTGACCAAGTTCATGCTTTGGATGATTGATGAAGAGG |
| KASP07253F2 | GAAGGTCGGAGTCAACGGATTTTGGATGATTGATGAAGAGA |
| KASP07253R | CTCATCTAACAAAGACCAAA |
| KASP0728F1 | GAAGGTGACCAAGTTCATGCTTCTTAGTGTATCATGAGCTT |
| KASP0728F2 | GAAGGTCGGAGTCAACGGATTTCTTAGTGTATCATGAGCTC |
| KASP0728R | CCAACTCGAGCTAGCTCGTG |
| KASP08096F1 | GAAGGTGACCAAGTTCATGCTTGCTCATCGTTTCGAACCGT |
| KASP08096F2 | GAAGGTCGGAGTCAACGGATTTGCTCATCGTTTCGAACCGC |
| KASP08096R | GGTGCAATTCAGCTTTATCG |
| KASP09121F1 | GAAGGTGACCAAGTTCATGCTTACACGTGGTGTTGTGTGTG |
| KASP09121F2 | GAAGGTCGGAGTCAACGGATTTACACGTGGTGTTGTGTGTA |
| KASP09121R | AAACTTAGGCTCTAATCTAA |
| KASP09150F1 | GAAGGTGACCAAGTTCATGCTCTTGCTGCTGCCCCGCCGTC |
| KASP09150F2 | GAAGGTCGGAGTCAACGGATTCTTGCTGCTGCCCCGCCGTT |
| KASP09150R | TTGTTGGCCACCCCCAACAC |
| KASP0921F1 | GAAGGTGACCAAGTTCATGCTGGGAGAGGCCGTGGTGGTCA |
| KASP0921F2 | GAAGGTCGGAGTCAACGGATTGGGAGAGGCCGTGGTGGTCG |
| KASP0921R | TCACCACGTGTGCCTCTGCCTCTT |
| NGSP11F | TGACACGCCACAGTCCAAGACGAGCAGT |
| 210QCF | AAGGGAGTTGAGAGTAGAAAAAA |
